# Prevalence, Antibiotic-Resistance, and Growth Profile of *Vibrio* spp. Isolated From Fish and Shellfish in Subtropical-Arid Area

**DOI:** 10.3389/fmicb.2022.861547

**Published:** 2022-04-06

**Authors:** Tarfa Abdalla, Hind Al-Rumaithi, Tareq M. Osaili, Fayeza Hasan, Reyad S. Obaid, Aisha Abushelaibi, Mutamed M. Ayyash

**Affiliations:** ^1^Department of Food Science, College of Agriculture and Veterinary Medicine, United Arab Emirates University (UAEU), Al Ain, United Arab Emirates; ^2^Department of Clinical Nutrition and Dietetics, College of Health Sciences, University of Sharjah, Sharjah, United Arab Emirates; ^3^Sharjah Institute for Medical Research, University of Sharjah, Sharjah, United Arab Emirates; ^4^Department of Nutrition and Food Technology, Faculty of Agriculture, Jordan University of Science and Technology, Irbid, Jordan; ^5^Campus Director at Higher Colleges of Technology, Dubai, United Arab Emirates

**Keywords:** *Vibrio* spp., antibiotic resistance, pH, salinity, temperature, prevalence

## Abstract

The study aimed to determine the prevalence of different species of *Vibrio* spp. in fish and shellfish sold in subtropical-arid countries (United Arab Emirates). It also examined the antimicrobial resistance of the isolated species and their growth behavior upon *in vitro* environmental changes concerning temperature, pH, and salinity. The prevalence of *Vibrio* spp. in fish and shellfish samples, was 64.5 and 92%, respectively. However, *Vibrio parahemolyticus* were detected in a mere 7.5 and 13.0% of the samples, respectively. On the other hand, *Vibrio mimicus* was detected in 1.5 and 8.5% of the samples, respectively. None of the six antibiotics studied except for Sulfamethoxazole-trimethoprim were effective against fish *Vibrio* spp. isolates. On a similar note, three antibiotics, namely Penicillin, Daptomycin, and Vancomycin, were ineffective against the shellfish isolates. The growth of the microorganisms did not show any significant trend with changes in pH and salinity. The optimum temperature for *Vibrio* spp. growth was observed to be 37°C.

## Introduction

*Vibrio* spp. are Gram-negative halophile and mesophile bacteria (Broberg et al., 2011). Different species of *Vibrio* exist, such as *V*. *parahaemolyticus* and *V. vulnificus*, to name a few (Chowdhury et al., 2004; Bonnin-Jusserand et al., 2019). *Vibrio cholerae* causes cholera which could be fatal (CDC, 2018). The symptoms associated with a *Vibrio* spp. infection are usually gastrointestinal, commonly expressed as watery diarrhea, abdominal cramping, nausea, vomiting, and fever (CDC, 2019d). The symptoms may show within 24 h of ingestion and may last up to 3 days (CDC, 2019c). Immunocompromised patients or those with a pre-existing condition are at a greater fatality risk (CDC, 2019c). The bacteria could also be resistant to multiple antibiotics making their treatment in a human infection difficult (Alsalem et al., 2018).

*Vibrio* spp. are good swimmers and can attach to other organisms living in the water (McCarter, 1999). Thus, a contaminated water body could potentially infect all its fish inhabitants (Kim and Lee, 2017). Multiple deadly outbreaks associated with *Vibrio* spp. have been observed worldwide (CDC, 2018; WHO, 2021). In a developed country like the United States, Vibriosis causes 80,000 illnesses and about 100 deaths every year (CDC, 2019b). There is a very high possibility that the actual number of outbreaks is higher than the reported figures (WHO, 2021).

Oysters feed by filtering water; hence, there is a chance that microorganisms concentrate in their bodies; thereby, they are at a higher risk of *Vibrio* spp. infection as compared to fish (CDC, 2019a). The prevalence of *V. parahaemolyticus* (from all the *Vibrio* spp. analyzed) in shellfish in Egypt ranged from 9.3 to 16.7% (Abd-Elghany and Sallam, 2013; Youssef et al., 2018). The contamination was even higher in Kuwait, with 78% of the seafood being contaminated with *Vibrio* spp. (Al-Mouqati et al., 2012). Raw contaminated seafood may contaminate other foods or surfaces in contact, like chopping boards, knives, etc. This cross-contamination puts other food items being prepared at the facility at risk, which may result in outbreaks, especially where the food is served to the masses, for instance, restaurants, and food catering organizations (Kramer et al., 2006). Moreover, slightly cooked or uncooked seafood delicacies may increase the risk of infection (CDC, 2019b).

The global fish and seafood trade value was estimated to be around USD 152 billion in 2017 (UNSTAD, 2019). The average seafood consumption *per capita* in the United Arab Emirates (UAE) is 29 kg/year (FAO, 2021). A total of 2,598 tonnes of fish worth 15 million USD were caught in UAE waters as of 2019 (SCAD, 2020). The fish production in the country is of substantial value, which could pose a major risk if contamination levels are high.

To the best of our knowledge, this is the first study to evaluate the microbiological safety of fish and shellfish in the UAE concerning *Vibrio* spp. Therefore, this study aimed to determine the prevalence of different species of *Vibrio* spp. in fish and shellfish sold in the local markets of the UAE. It also examined the antimicrobial resistance of the isolated species and their growth behavior upon *in vitro* environmental changes.

## Materials and Methods

### Sample Collection

Fresh most sold local 200 fish samples (spangled emperor, prang spotted, pearly goatfish, greater amberjack, and yellowstripe scad) and 200 shellfish (shrimp, oysters, crab, clam, and lobster) were purchased from local markets in four different emirates in the UAE (Al-Ain, Dubai, Fujairah, and Abu Dhabi). These emirates are housing the larger fish markets in the whole UAE. From each emirate, 50 fish and 50 shellfish samples were collected (10 samples per each above-mentioned type). The samples were purchased from June to September 2017 and transferred into sterile, sealable, labeled plastic bags. The samples were transported in dry ice to the food microbiology laboratory at the United Arab Emirates University (UAEU) for analysis.

### *Vibrio* spp. Isolation

Twenty-five grams of the flesh from fish and shellfish samples were homogenized in 225 ml alkaline peptone saline water (APSW, Hi Media®, Bombay, India) using a stomacher circular Unit 400 (Seward Ltd.®, London, United Kingdom) for 2 min at 260 rpm followed by incubation at 42°C for 8 h. Then, 10 ml of the incubated homogenate were streaked in duplicate on Thiosulfate-Citrate-Bile Salts-Sucrose Agar (TCBS Agar; Oxoid, Thermo Fischer Scientific) and Modified Cellobiose-Polymyxin B-Colistin Agar (mCPC Agar; APSW, Hi Media®, Bombay, India) followed by incubation at 37°C for 24 h. The suspected colonies were re-streaked on Tryptone Soy Agar (Oxoid, Thermo Fischer Scientific) supplemented with 3% Sodium Chloride (TSA + 3% NaCl) and incubated at 37°C for 24 h to obtain a pure isolate (Sujeewa et al., 2009).

### DNA Extraction

The isolated bacteria were grown individually in Tryptone Soy Broth (Oxoid, Thermo Fischer Scientific) supplemented with 3% NaCl (TSB + 3% NaCl) and incubated at 37°C for 24 h. The isolates’ DNA was extracted using a QIAGEN DNA extraction kit as per the manufacturer’s instructions.

### Confirmation of *Vibrio* spp. by Polymerase Chain Reaction

PCR assay was performed separately for general (*Vibrio* spp.) and specific (16S rRNA) genes of the suspected *Vibrio* spp. isolates. The process conditions were 35 cycles of amplification, denaturation at 94°C for 1 min, annealing at 58°C for 1 min, extension at 72°C for 1 min, and final extension at 72°C for 7 min. The reaction mixtures were resolved by electrophoresis in 2% agarose gel and visualized under UV light. Gel bands were compared with reference strains (*V. parahaemolyticus* DSM2172 and DSM19130, *V. vulnificus* DSM10143, and *V. mimicus* DSM19130). The reference strains were purchased from Leibniz-Institut DSMZ—Deutsche Sammlung von Mikroorganismen und Zellkulturen GmbH (Braunschweig, Germany). The listed primers in Table 1 were employed to identify the *Vibrio* spp. and strains. A 100 bp marker was employed (Sujeewa et al., 2009).

**Table 1:** Primers for *Vibrio* spp. identification.

Target bacterium	Primer sequence (5′ → 3′)
All *Vibrio* spp., 16S rRNA	F: CGGTGAAATGCGTAGAGAT R: TTACATGCGATTCCGAGTTC
*V. parahaemolyticus*	F: AAAGCGGATTATGCAGAAGCACTG R: GCTACTTTCTAGCATTTTCTCTGC
*V. mimicus*	F: CATTCGGTTCTTTCGCTGAT R: GAAGTGTTAGTGATTGCTAGAGAT
*V. cholerae*	F: AAGACCTCAACTGGCGGTA R: GAAGTGTTAGTGATCGCCAGAGT
*V. vulnificus*	F: GTCTTAAAGCGGTTGCTGC R: CGCTTCAAGTGCTGGTAGAAG

### Antibiotic Sensitivity of *Vibrio* spp. Isolates

Twenty-eight *Vibrio* isolates including 15 *V. parahaemolyticus*, 10 *V. vulnificus*, and 3 *V. mimicus* were used for antibiotic test. The isolates were inoculated into sterile TSB + 3% NaCl, which was then incubated at 37°C until turbidity (~16 h). Using a sterile cotton swab, the bacterium was inoculated on Muller Hinton Agar plates (Oxoid, Thermo Fischer Scientific). Antimicrobial susceptibility test disks (Oxoid, Thermo Fischer Scientific) of Penicillin G (10 iu), Vancomycin (2 mcg), Daptomycin (30 mcg), Ampicillin (10 mcg), Erythromycin (15 mcg), and Sulphamethoxazole/Trimethoprim (SXT; 25 mcg) were gently placed on the agar plates post which they were incubated at 37°C for 24 h. The inhibition zone was measured in millimeters (Yaashikaa et al., 2016).

### Analysis of Factors Affecting Growth and Survival of *Vibrio* spp.

#### Effect of Temperature on the Growth of *Vibrio* spp. Isolates

Quantities of 0.1 ml of the isolated *Vibrio* spp. cultures were inoculated in sterilized nutrient broth (Oxoid, Thermofischer scientific) and incubated for 20–24 h at different temperatures (25, 37, and 45°C). Appropriate serial dilutions using Peptone water were then prepared, and the tubes were re-incubated for 20–24 h at 37°C. The viable counts were determined using a spectrophotometer adjusted to 620 nm at regular time intervals (Yaashikaa et al., 2016).

The growth rate was calculated in comparisons with Optical Density (OD) at time 0 h (*t*0) and time the specimen was analyzed (*t*):


$$\%\mathrm{of}\;\mathrm{growth}\;\mathrm{rate}=\frac{OD_{t}-OD_{t0}}{OD_{t}}\times 100$$

#### Effect of pH on the Growth of *Vibrio* spp. Isolates

Quantities of 0.1 ml of the isolated *Vibrio* spp. cultures were inoculated in nutrient broth adjusted to a pH of 3, 5, and 7 using 0.1 N HCl and incubated for 20–24 h at 37°C. After that, appropriate serial dilutions using Peptone water were made, and the tubes were re-incubated at 37°C for 24 h. The viable count of bacteria was determined with the help of a spectrophotometer adjusted to 620 nm at regular time intervals (Yaashikaa et al., 2016). The growth rate was calculated in comparison with OD at time 0 h.

#### Effect of Salinity on the Growth of *Vibrio* spp. Isolates

NaCl was added at various concentrations (0.5, 1.0, and 2.0%; Yang et al., 2010) to nutrient broth, after which the pH was adjusted to 8.5 using Sodium Hydroxide (NaOH, 0.1 N). The test tubes containing the mixture were then autoclaved. The tubes were then inoculated with 0.1 ml of fresh *Vibrio* spp. isolates, and incubated for 20–24 h at 37°C. Appropriate serial dilutions were performed using Peptone water for each concentration. Growth of isolates was observed by measuring the absorbance using a spectrophotometer adjusted to 620 nm at regular time intervals (Yaashikaa et al., 2016). The growth rate was calculated compared to OD at time 0 h as described in the section “Effect of temperature on the growth of *Vibrio* spp. isolates.”

### Statistical Analysis

Growth profile data in triplicate were subjected to the ANOVA using a general linear model (GLM). Mean comparisons were performed using Duncan’s multiple range test to compare significant differences between means for all analyses. Statistical analysis was carried out using Statistical Package for the Social Sciences (SPSS®, Version 21). Values are expressed as mean ± SD. The contour plots were performed using Minitab v21 (Pennsylvania, United States). The regression equations used were:

For fish isolates, OD = −1.203 + 0.4149 Salt + 0.1402 pH + 0.00745 Temp.

For shellfish isolates, OD = −0.341 + 0.2859 Salt + 0.1119 pH − 0.00590 Temp.

## Results and Discussion

### Isolation of *Vibrio* spp. in Fish and Shellfish Samples

Fish can harbor various microorganisms like *Listeria monocytogenes*, *Yersinia* spp., *Salmonella* spp., and *Clostridium botulinum* (Novoslavskij et al., 2016). The microorganisms in fish can use it as a nutrient medium and hydrolyze proteins to form biogenic amines (Lou et al., 2021). However, one notorious pathogen of great public health concern that fish can harbor is the *Vibrio* spp. (Novoslavskij et al., 2016). The cases of Vibriosis are on the rise even in developed countries like the United States (Baker-Austin et al., 2018).

In this study, eighth respect to fish and shellfish samples, the results showed that a total of 129 (64.5%), 184 (92%) were *Vibrio* spp., positive, respectively. The high prevalence of *Vibrio* spp. could be attributed to ecological contamination like that of the feed or to the water salinity and temperatures (Blanch et al., 2009; Baker-Austin et al., 2018). The UAE follows strict practices to prevent marine pollution; thereby contamination through this means is highly unlikely. High seawater rising temperatures attributable to the worldwide phenomenon of global warming could be a possible explanation (Baker-Austin et al., 2018). However, the current prevalence rates observed in this study may not necessarily be a cause of concern as the country has not witnessed any major outbreak of Cholera or Vibriosis (to the best of our knowledge; SCAD, 2020). *Vibrio* spp. is indigenous to marine waters (Hsiao and Zhu, 2020). Furthermore, not all variants of *Vibrio* spp., are considered as pathogenic (Lopez-Joven et al., 2015; Song et al., 2017).

The comparative higher prevalence in shellfish than fish is understandable because actively growing clear shellfish particles from water at rates ranging from 1 to 4 L/h (Rice, 2001; CDC, 2019a). It is thereby possible that during this filtration step, the *Vibrio* spp., present in the water body, is retained within the shellfish body (Rice, 2001; CDC, 2019a). A meta-analysis did indicate a general trend of higher *Vibrio* spp., contamination in oysters and clams compared to fish (Odeyemi, 2016).

Moreover, Fish is usually consumed after applying a heat treatment, which is expected to destroy any pathogenic *Vibrio* spp. (USDA, 2020). Further analysis regarding the prevalence of virulent strains is needed for better comprehension.

The prevalence of *Vibrio* spp., isolates in shellfish from Egypt, was observed to be 33% (Abd-Elghany and Sallam, 2013). A study in Iran reported a 17.1% prevalence of *Vibrio* spp., in shrimp samples (Asgarpoor et al., 2018). A higher population of *Vibrio* spp., in shrimp samples purchased from wet markets (5.04–6.34 log CFU/ml) compared to supermarkets (4.21–4.43 log CFU/ml) was observed in Malaysia (Letchumanan et al., 2015). Another study conducted in Iran revealed 26.8% of the examined fish samples were *Vibrio* spp., positive (Raissy et al., 2014). The prevalence of *Vibrio* spp., varies based on water temperature, level of salinity, season, water depth, and total suspended solids (Akoachere and Mbuntcha, 2014; Kokashvili et al., 2015; Lopez-Joven et al., 2015; Williams et al., 2017).

### Molecular Identification of *Vibrio* spp. Isolates in Fish and Shellfish Samples

**Figure 1:**
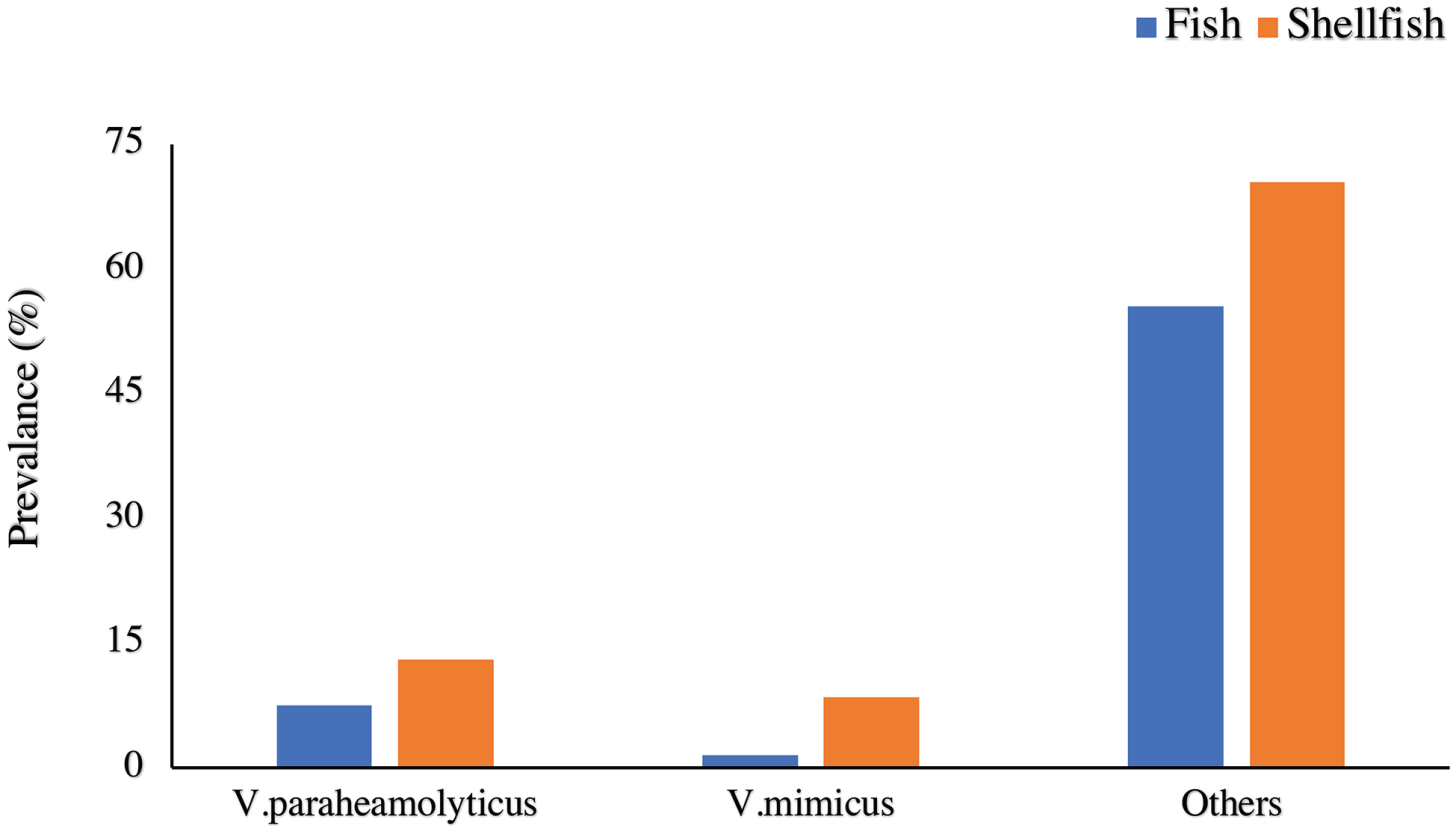
Prevalence of different *Vibrio* spp., in Fish and Shellfish.

In fish, the majority species prevalence was *Vibrio parahemolyticus* and *V*. *mimicus* (7.5 and 1.5%, respectively; Figure 1). A similar result was observed in shellfish samples, with 13 and 8.5% prevalence, respectively (Figure 1). *Vibrio vulnificus* was found in fish (5%), but it was not present in the shellfish samples. A study conducted in the Suez Canal area (Egypt) reported an overall *V. parahaemolyticus* prevalence of 9.3% in shellfish (Youssef et al., 2018). The entire prevalence of the above-mentioned species does not need to be pathogenic. The virulence genes present in the bacteria are a cause of concern in terms of public health (Hasan et al., 2010; Gennari et al., 2012; Lopez-Joven et al., 2015). This study is limited to the typification of the species, and further research is needed to analyze the pathogenicity of these strains. The strain pathogenicity depends on factors like the presence of enzymes, such as urease, lipase, gelatinase, and hemolysin or the adhesiveness potential (Baffone et al., 2001).

The percentage of *V. parahaemolyticus* in shellfish harvested from Turkey was observed to be 0.8% (Colakoglu et al., 2006). Amongst all the *Vibrio* spp., studied, some studies indicate that *V*. *alginolyticus* was present in the greatest amount of samples (Gopal et al., 2005; Colakoglu et al., 2006; Yücel and Balci, 2010), while others were indicating a higher prevalence of other strains (*Vibrio cholerae*, *Vibrio communis*; Adebayo-Tayo et al., 2011; Amalina et al., 2019).

PCR was used for the molecular identification of the *Vibrio* spp., positive isolates. The presence of *Vibrio* spp. was confirmed by using both general and *Vibrio* spp., specific sequences. In this study, the presence of *Vibrio* spp., in shellfish samples were atypical in different locations. 16S rRNA is present in all *Vibrio* spp., isolates and could be used as a marker gene for specific detection of this bacterium (Kim et al., 1999).

### Antibiotic Resistance of *Vibrio* spp. Isolates

Various antibiotic-resistant *Vibrio* spp., isolates have been reported worldwide (Baker-Austin et al., 2009; Kitaoka et al., 2011; Jun et al., 2012; Kang et al., 2017; Kurdi Al-Dulaimi and Ariffin, 2019). These bacteria develop the resistance through various mechanisms, including the development of multidrug efflux pumps, horizontal gene transfer, plasmid conjugation, or just simple random mutations (Kitaoka et al., 2011). If the antibiotic-resistant bacteria get transferred to humans *via* the food chain, it will create difficulties in disease treatment (Igbinosa, 2016).

With respect to *V*. *parahemolyticus* and *V*. *mimicus* isolated from fish in this study, none of the studied antibiotics (penicillin g, daptomycin, vancomycin, ampicillin, and erythromycin) were effective except for SXT (40% resistance). On a similar note, in shellfish isolates, treatment with penicillin, daptomycin, and vancomycin proved to be futile (Table 2). Erythromycin and SXT were more effective against *V*. *parahemolyticus* isolates (62.0 and 8.0% were resistant, respectively) compared to *V. mimicus* (94 and 12.0% were resistant, respectively) in shellfish (Table 2). The difference in resistance to antibiotics in fish and shellfish isolates despite belonging to the same strain could be because the immune response varies based on fish type—those strains isolated from fish with higher immune responses may have developed mechanisms for enhanced survival compared to fish with lower immunity (Smith et al., 2019). Further studies are needed to confirm this hypothesis. Similarly, any previous exposure to antibiotics may also encourage survival (Smith et al., 2019). Other modes of treatment could decrease the prevalence of the *Vibrio* spp., such as ultrasound, low-temperature treatment, and the use of ozone and saline (Zhou et al., 2002). Moreover, the fish/shellfish are expected to be marinated prior to consumption. The use of sugar, vinegar, lemon juice, or citric acid has been associated with decreasing the contamination of *Vibrio* spp., in fish and shellfish, respectively (Borazjani et al., 2003; Ibrahim et al., 2018).

**Table 2:** Percentage of *Vibrio* spp., isolates resistant to antibiotics.

Antibiotic	Concentration	Type	*V. parahemolyticus*	*V. mimicus*	Other *Vibrio* species
Penicillin G	1 IU	Fish	100	100	100
		Shellfish	100	100	41
Daptomycin	2 mcg	Fish	100	100	100
		Shellfish	100	100	89
Vancomycin	30 mcg	Fish	100	100	100
		Shellfish	100	100	74
Ampicillin	10 mcg	Fish	100	100	100
		Shellfish	27	6	10
Erythromycin	15 mcg	Fish	100	100	100
		Shellfish	62	94	21
SXT	25 mcg	Fish	40	40	33
		Shellfish	8	12	2

SXT, sulfamethoxazole-trimethoprim; IU, international units; and mcg, microgram.

In accordance with our observation, a previous study on *Vibrio* spp., isolates from cockles and clams showed resistance toward Penicillin (93%), Ampicillin (70%), Cephalothin (65%), Clindamycin (66%), Vancomycin (64%), and Erythromycin (51%; Kurdi Al-Dulaimi and Ariffin, 2019). A study conducted on 44 *V*. *parahaemolyticus* isolates from oysters in Korea revealed 90.9, 86.4, and 75.0% of the isolates being resistant to Vancomycin, Ampicillin, and Streptomycin treatment, respectively (Kang et al., 2017). Another study in Korea reported all 19 isolates of *V*. *parahaemolyticus* obtained from seafood to be resistant to more than four commercial antibiotics (Jun et al., 2012). Other studies also report similar observations (Baker-Austin et al., 2009; Lopatek et al., 2015). On the other hand, an assessment of the antimicrobial susceptibility profile of *V*. *parahaemolyticus* isolated from short mackerels (*Rastrelliger brachysoma*) in Malaysia revealed majority of the isolates were highly susceptible to Ampicillin Sulbactam, Meropenem, Ceftazidime, and Imipenem (Tan et al., 2017). Treatment with Ampicillin/Sulbactam or Chloramphenicol was effective against around 95% of the *V*. *parahaemolyticus* strains isolated from shrimps in Malaysia (Letchumanan et al., 2015).

In contrast to antibiotic therapy, acid electrolyzed ice water decreased *Vibrio* spp., populations by about 3% (He et al., 2022). Meanwhile, a synergistic antimicrobial effect on the microbiota of fish was observed when it was vacuum packaged with a coating of gelatin composed of grape seed extract. This strategy may also be useful on pathogenic species like *Vibrio* spp. (Zhao et al., 2021).

### Factors Affecting the Growth Rate of *Vibrio* spp. Isolates of Shellfish

#### Effect of Salt Concentration on the Growth Rate of *Vibrio* spp. Isolates

Salt in the form of NaCl has been observed to enhance the formation of wrinkle colonies and pellicle in *Vibrio* spp. (Marsden et al., 2017). The formation of wrinkled colonies happens at the earlier stages of biofilm formation (Ray et al., 2012). Even at high salt concentrations, the rapid growth of bacteria is a cause of concern as it is a very common/cost-effective preservative method. The *Vibrio* spp., isolates from fish and shellfish in this study were thereby tested for their ability to grow in the presence of varying salt concentrations. After 16 h of storage, at a salt concentration of 0.5%, the growth rates of the *Vibrio* isolates (*n* = 28) ranged from 58.9 to 92.5% and 51.7 to 83.3% in fish and shellfish, respectively. Increasing the salt concentration to 1% resulted in growth rates of 44.8–82.3 and 34.8–72.3%, respectively. Furthermore, a salt concentration of 2% resulted in growth rates ranging from 56.6 to 87.1% and 38.6 to 81.0%, respectively. Great variation among strains was observed with no specific trend (Supplementary Figure S1).

The literature regarding the impact of NaCl on *Vibrio* spp., growth is bifurcated. A study conducted on *V*. *parahaemolyticus* and *V*. *vulnificus* showed that the bacteria reached a viable-but-nonculturable (VBNC) state when the concentration of NaCl was elevated up to a level of 30% (Yoon et al., 2017). Decreased water salinity levels have also been associated with increased concentration of *V*. *parahaemolyticus* in shrimp aquaculture systems (Bauer et al., 2021). The toxicity of *Vibrio* spp., grown in 1% NaCl conditions, was greater than a 3% concentration (Whitaker et al., 2010). On the other hand, the growth of *V*. *parahaemolyticus* in 1% NaCl being significantly less when compared to growth in 3% NaCl has also been reported (Whitaker et al., 2010). About 80% of the *Vibrio* spp., extracted from fish and prawns, could grow in salt concentrations ranging from 1.5 to 3.5% (Surendran et al., 1983). The variance in observation could be explained. *Vibrio* spp. can be classified as moderate halophiles (Surendran et al., 1983). They do need salt for their survival/growth (Fujiwara-Nagata and Eguchi, 2004); however, increasing the concentration beyond a certain limit seems detrimental (Yoon et al., 2017; Bauer et al., 2021). A study conducted on *V*. *alginolyticus* indicated that the highest growth of the microbe was observed at 3% NaCl solution, increasing the concentration to 6% or decreasing it to 0.5% lowered growth (Farid and Larsen, 1981). Sodium is essential for *Vibrio* spp., for growth and starvation survival mechanisms (Fujiwara-Nagata and Eguchi, 2004). To overcome salt stress, the microbe remodels its outer membrane proteins to keep an intact cell membrane (Yang et al., 2010). As can be seen from the previous studies, the ability of each strain to use sodium for its growth or to protect itself from the harsh environment is expected to vary, and this does explain the no consistent trend seen in our results.

#### Effect of pH on the Growth Rate of *Vibrio* spp. Isolates

The acidity or alkalinity of the surrounding environment exerts antimicrobial action on the bacterial cell by affecting the proteins involved in cell membrane maintenance and motility function (Hommais et al., 2002). Furthermore, the pH affects the ability of the *Vibrio* spp., cell in terms of its ability to resist drugs and interferes with its biofilm formation (Hommais et al., 2002). The *Vibrio* spp., isolates from fish and shellfish of this study were tested for their ability to withstand varying pH conditions. The growth rate (depending on the isolate) at pH = 3.0 ranged from 1.5 to 60.1 and 1.8 to 65.0% in fish and shellfish, respectively. At a pH of 5.0, the growth rate range was 44.5–87.1 and 8.7–67.4%, respectively. At neutral pH = 7.0, the growth rates for fish and shellfish were from 25.6 to 81.6 and 38.6 to 76.2%, respectively (Supplementary Figure S2).

The *V*. *cholerae* and *V*. *parahaemolyticus* isolated from prawn (*Penaeus monodon*) were reported to grow well at pH 5.0 and 7.0. Increasing the pH to 9.0 or reducing it to 3.0 decreased the growth of *V*. *cholerae* and *V*. *parahaemolyticus* isolates (Yaashikaa et al., 2016). Another study indicated the ability of *Vibrio* spp., to survive broad spectra of pH ranging from 5.2 to 9.2 (Beuchat, 1973). Our study results are compatible with the above literature, as the minimal growth rate at pH 3.0 is lower by a good degree when compared to a pH of 5.0 or 7.0 (Supplementary Figure S2).

#### Effect of Temperature on the Growth Rate of *Vibrio* spp. Isolates

The temperature of the environment alters the cell membrane fluidity and thereby impacts pathogen survival (Kandušer et al., 2008). At a temperature of 25°C, the growth rate of *Vibrio* spp., in fish and shellfish ranged from 47.5 to 82.8 and 8.4 to 80.5%, respectively. Increasing the temperature to 37°C resulted in growth rates ranging from 54.5 to 84.7 and 34.0 to 80.9%, respectively. At 45°C, the growth rates were 19.2–75.3 and 27.7–76.1%, respectively. The results indicate that the optimum temperature for the *Vibrio* spp., growth was at 37°C (Supplementary Figure S3).

In a previous study, *V*. *parahemolyticus* populations in fresh seafood were 33.4%, while the prevalence in frozen and iced samples was observed to be 14.9% (Yang et al., 2008). In another study, *V*. *parahaemolyticus* grew well at 15, 25, and 35°C; however, a lower growth rate was observed at 5°C (Wang et al., 2018). *Vibrio parahaemolyticus* in live clams held at 28°C multiplied rapidly while no significant growth was observed and 4 and 15°C (Lopez-Joven et al., 2018). In Oyster meat stored at 16°C, *V*. *vulnificus* populations showed a maximum increase by 1.5 log CFU/g, while a storage at 36°C, resulted in a maximum increase by 2.8 log CFU/g, respectively (Kim et al., 2012). In Oyster slurry, *V*. *parahaemolyticus* growth was not observed at 10 and 15°C. *Vibrio parahemolyticus* was observed to have an optimum growth rate at temperatures ranging from 37 to 39°C, increasing or decreasing the temperature beyond 8.3 and 45.3°C resulting in hampering their growth (Miles et al., 1997). Our results are in accordance with the above-mentioned studies.

#### Regressions Between the Growth Factors

**Figure 2:**
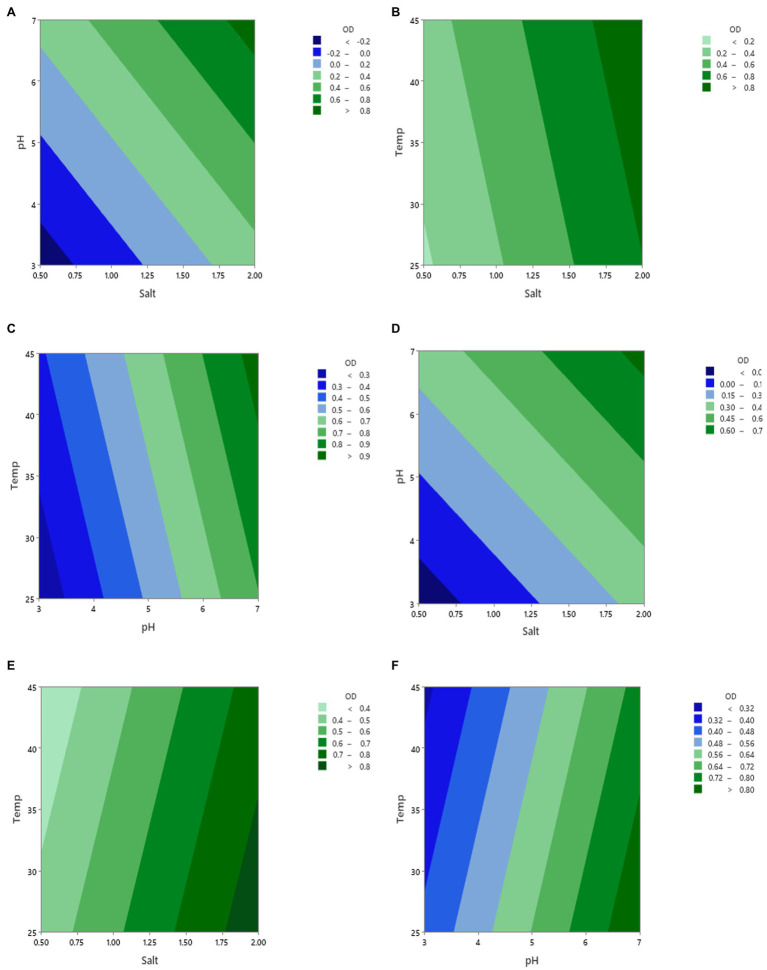
Contour plots of the growth (OD) of vibrio spp. isolated from fish **(A–C)** and shellfish **(D–F)** during changing three factors (pH, salt, and temperature) and incubation for 16 h. The fixed values of each factor were 2.0 for salt, 7.0 for pH, and 37°C for temperature.

Figure 2 displays the contour plots of the effect of the three factors pH, salt, and temperature on the growth (OD) of *Vibrio* spp., from fish (A–C) and shellfish (D–F) after 16 h of incubation. One factor was fixed when the other two factors were changed. As can be seen from the figure, the variation in the contour ranges was pronounced when pH was changed. This implies that pH had a greater influence on the *Vibrio* spp., growth than salt and temperature. This suggests that using acidulant agents would be an efficient approach to preserve fish and shellfish products in addition to temperature.

## Conclusion

To the best of our knowledge, our study is the first comprehensive report regarding the prevalence, growth characteristics, and antibiotic susceptibility of *Vibrio* spp., isolates from fish and shellfish samples in the UAE. The prevalence of *V. parahemolyticus* and *V. mimicus* was low. *V*. *vulnificus* was found only in a minor portion of fish samples. A definitive conclusion cannot be made about the risk they pose. This is because the presence of virulence genes present in the microorganism defines its pathogenicity. The analysis at the gene level was not conducted in this study. However, fish and shellfish are usually given a heat treatment prior to consumption in our region, thereby posing less risk. The impact of environmental growth conditions was observed to vary greatly based on the strain. SXT was determined to be the most effective antibiotic in the treatment of *V. parahemolyticus* and *V. vulnificus* isolates from fish, while both Ampicillin and SXT were effective in shellfish.

## Data Availability Statement

The original contributions presented in the study are included in the article/Supplementary Material; further inquiries can be directed to the corresponding authors.

## Author Contributions

MA, TO, and AA: conceptualization. TA and HA-R: methodology, investigation, and data curation. MA and TO: validation. TA, HA-R, and MA: formal analysis. MA: resources, supervision, project administration, and funding acquisition. TA, HA-R, TO, and FH: writing—original draft preparation. MA, TO, and RO: writing—review and editing. TA, HA-R, TO, and MA: visualization. All authors contributed to the article and approved the submitted version.

## Funding

The authors are thankful to the United Arab Emirates University for funding this project.

## Conflict of Interest

The authors declare that the research was conducted in the absence of any commercial or financial relationships that could be construed as a potential conflict of interest.

## Publisher’s Note

All claims expressed in this article are solely those of the authors and do not necessarily represent those of their affiliated organizations, or those of the publisher, the editors and the reviewers. Any product that may be evaluated in this article, or claim that may be made by its manufacturer, is not guaranteed or endorsed by the publisher.
