# Supplementary material for: Prevalence, Antibiotic-Resistance, and Growth Profile of Vibrio spp. Isolated From Fish and Shellfish in Subtropical-Arid Area
Source: Front Microbiol. 2022 Apr 6;13:861547. doi: 10.3389/fmicb.2022.861547 (PMC9019552; doi:10.3389/fmicb.2022.861547)
Supplement: Supplementary file 1 [file Data_Sheet_1.PDF]

Supplementary Materials

Prevalence, antibiotic-resistance and growth profile of *Vibrio* spp., isolated from fish and shellfish in subtropical-arid area

Tarfa Abdalla[1#], Hind Al-Rumaithi[1#], Tareq M. Osaili[2,3,4*], Fayeza Hasan[3], Reyad S. Obaid[2], Aisha Abushelaibi[5], Mutamed M. Ayyash[1*]

[1] Department of Food Science, College of Agriculture and Veterinary Medicine, United Arab Emirates University (UAEU), Al Ain, UAE

[2] Department of Clinical Nutrition and Dietetics, College of Health Sciences, University of Sharjah, Sharjah P.O. Box 27272, United Arab Emirates.

[3] Sharjah Institute for Medical Research, University of Sharjah, Sharjah P.O. Box 27272, United Arab Emirates.

[4] Department of Nutrition and Food Technology, Faculty of Agriculture, Jordan University of Science and Technology, P.O. Box 3030, Irbid 22110, Jordan.

[4] Campus Director at Higher Colleges of Technology, Dubai, UAE.

# Both authors contributed equally to the paper.
*Corresponding author:
Dr. Mutamed M. Ayyash mutamed.ayyash@uaeu.ac.ae
Prof. Tareq M. Osaili tosaili@sharjah.ac.ae

Growth rate of *Vibrio* isolates at 0.5% NaCl

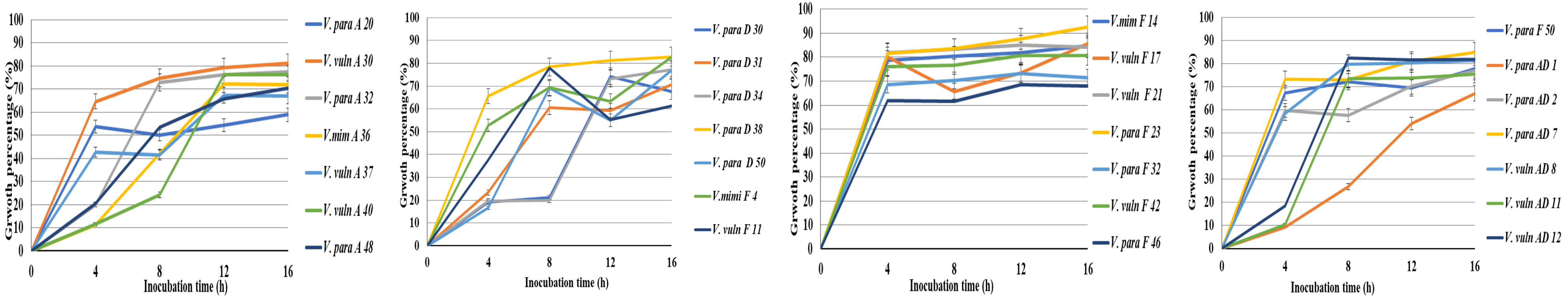

Growth rate of *Vibrio* isolates at 1.0% NaCl

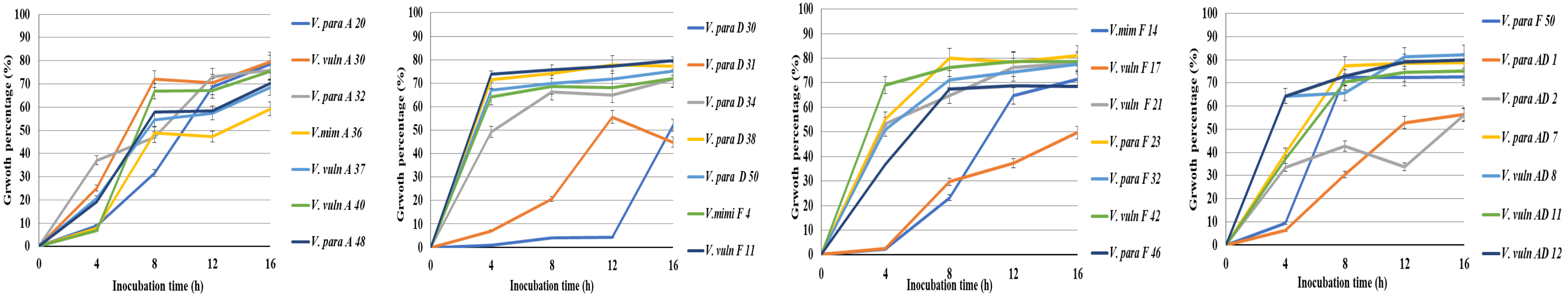

Growth rate of *Vibrio* isolates at 2.0% NaCl

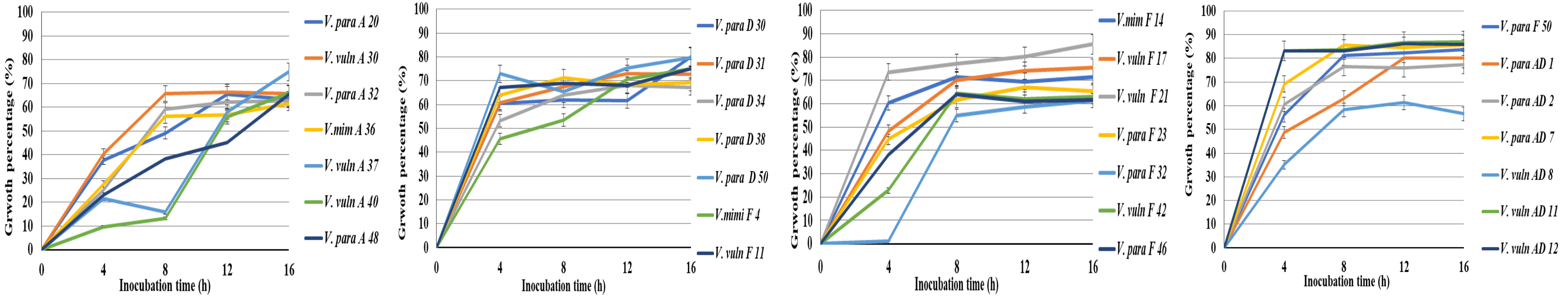

Figure S1: Growth rate of Vibrio isolates at 0.5, 1.0%, and 2.0% NaCl. Values are expressed as average of 3 replicates ± SD

Growth rate of *Vibrio* isolates at pH 3.0.

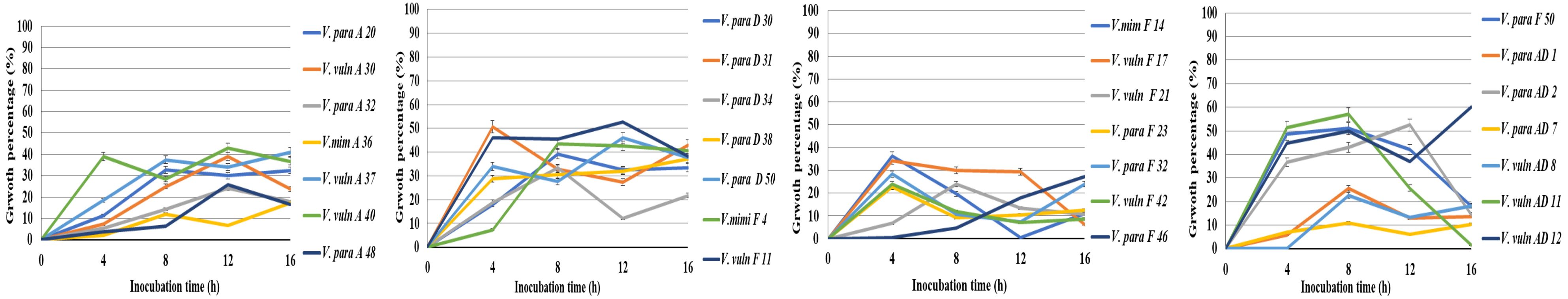

Growth rate of *Vibrio* isolates at pH 5.0

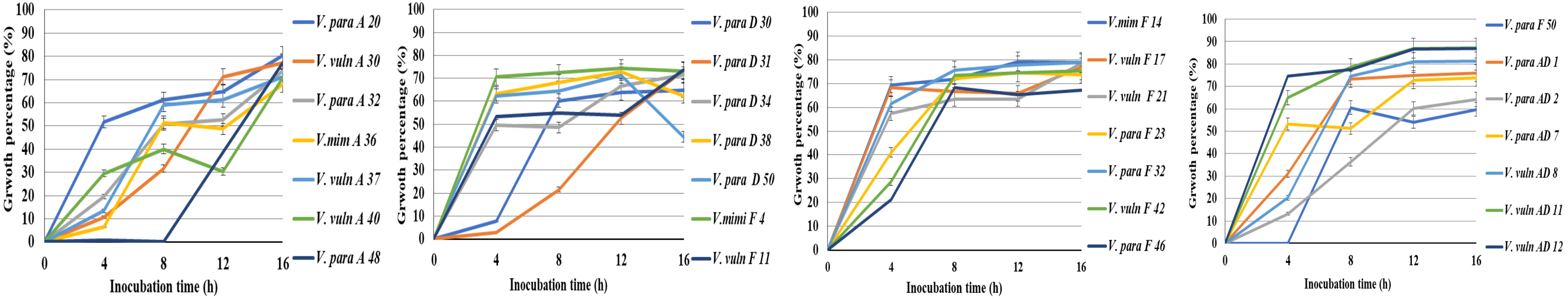

Growth rate of *Vibrio* isolates at pH 7.0

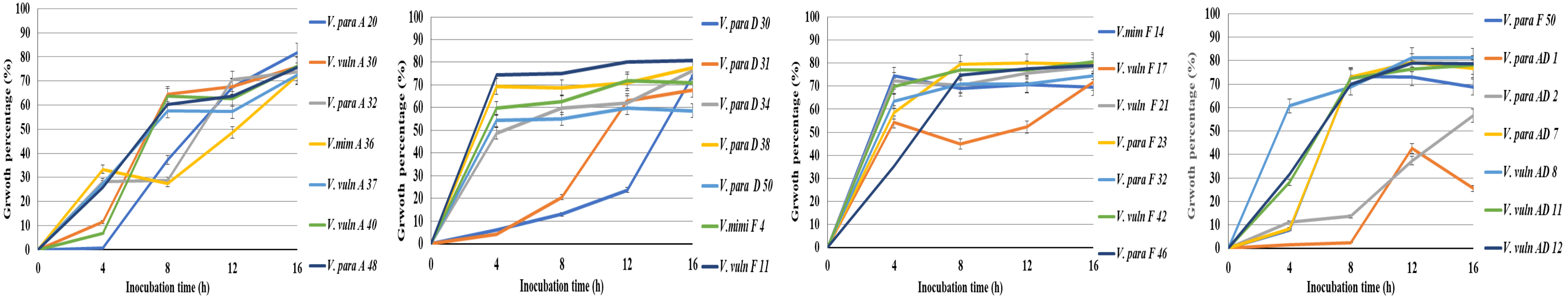

Figure S2: Growth rate of *Vibrio* isolates from at pH 3.0, 5.0, and 7.0. Values are expressed as average of 3 replicates ± SD

Growth rate of *Vibrio* at 25°C

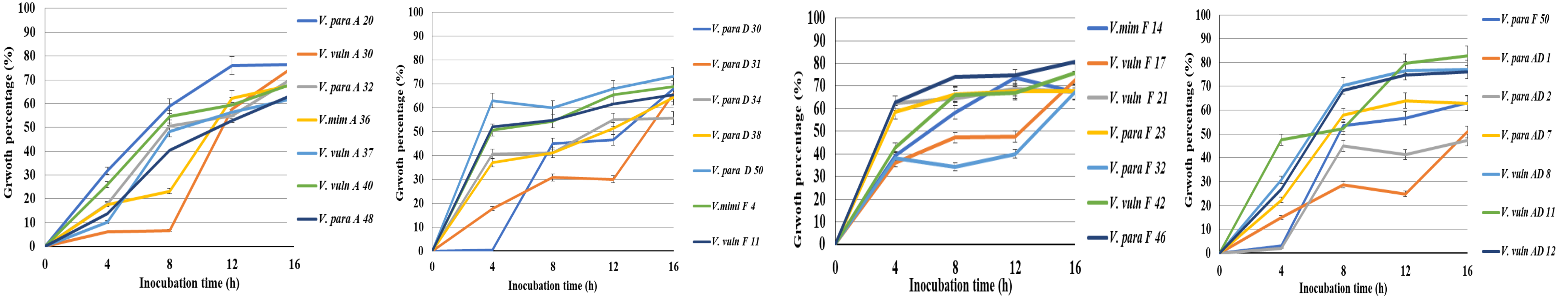

Growth rate of *Vibrio* at 37°C

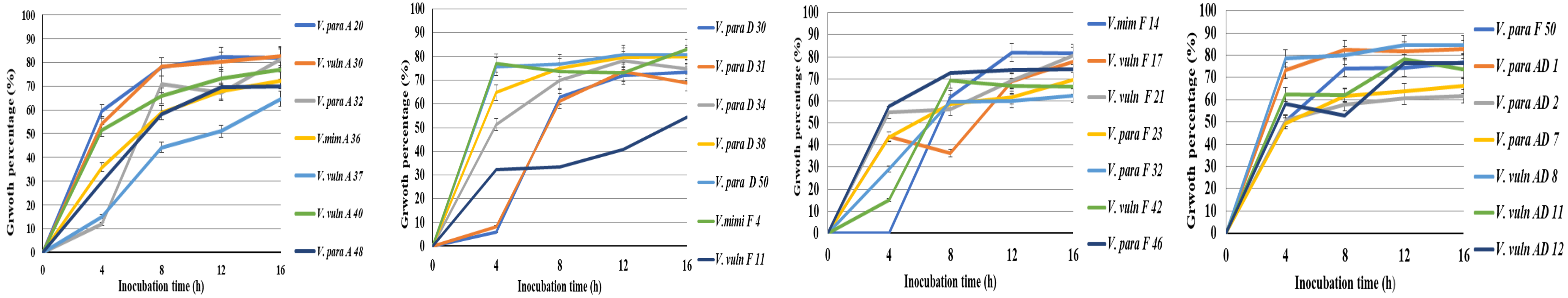

Growth rate of *Vibrio* at 45°C

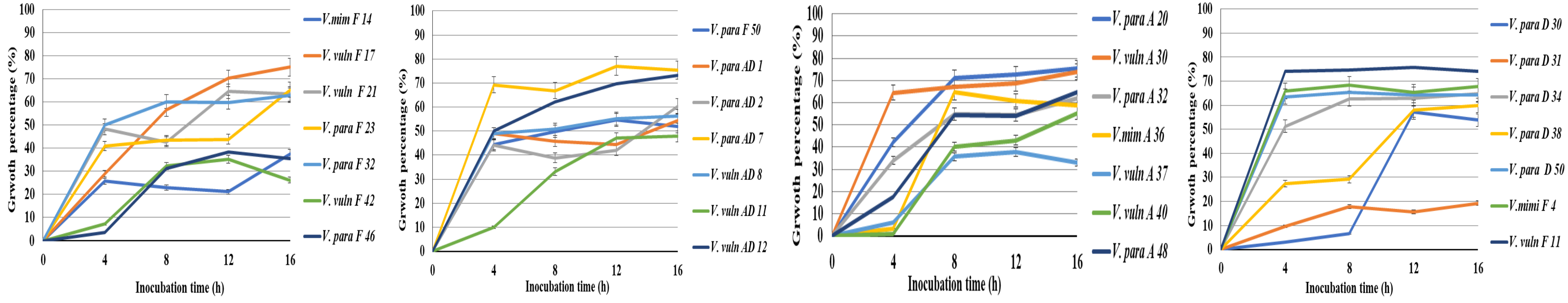

Figure S3: Growth rate of Vibrio isolates at 25°C, 37°C, and 45°C. Values are expressed as average of 3 replicates ± SD
